# A Generalized Quantum-Inspired Decision Making Model for Intelligent Agent

**DOI:** 10.1155/2014/240983

**Published:** 2014-03-17

**Authors:** Yuhuang Hu, Chu Kiong Loo

**Affiliations:** Advanced Robotic Lab, Department of Artificial Intelligence, Faculty of Computer Science & Information Technology, University of Malaya, 50603 Kuala Lumpur, Malaysia

## Abstract

A novel decision making for intelligent agent using quantum-inspired approach is proposed. A formal, generalized solution to the problem is given. Mathematically, the proposed model is capable of modeling higher dimensional decision problems than previous researches. Four experiments are conducted, and both empirical experiments results and proposed model's experiment results are given for each experiment. Experiments showed that the results of proposed model agree with empirical results perfectly. The proposed model provides a new direction for researcher to resolve cognitive basis in designing intelligent agent.

## 1. Introduction

Decision making model is crucial to build successful intelligent agent. Therefore, study of decision making model plays a key role in order to improve performance of intelligent agent. Traditionally, decision making model is represented and implemented by employing Bayesian or Markov process [1, 2]. However, traditional methods usually introduce biases problems in designing stage. And many empirical findings in cognitive science in recent years have indicated that human usually violates “rational decisions” which are produced by traditional methods [3–6]. Hence, many researchers in cognitive science, psychology, neuroscience, and artificial intelligence have proposed different explanations to complete traditional methods [7–9]. However, none of the explanations is able to resolve all human's violations of “rational decisions” completely.

There are two major violations of “rational decision” found in previous studies: “sure thing principle” and “order effects.” The sure thing principle claims that human should prefer *A* over *B* if *A* is always better than *B* in world *W*. This principle was tested by Tversky and Shafir [10] in a simple two-stage gambling experiment and showed violation to the principle. The order effect argues that human's decision pattern violates a fundamental requirement of classical probability theory: Pr(*A*∩*B* | *C*) = Pr(*B*∩*A* | *C*) which implies Pr(*C* | *A*∩*B*) = Pr(*C* | *B*∩*A*) according to Bayesian rule [9]. The violations of “rational decision” are recognized as “wrong decisions” or “stupid decisions” in some game theorists' perspective. For example, the violation of the sure thing principle is an obvious wrong decision theoretically. However, people usually choose differently even though they completely understand the risks and benefits in the certain scenario such as [10]. And the order effect challenges the classical theory even more fundamentally. The commutative property is not followed in human decision making process, which means the analyses based on classical probability theory introduce serious bias of modeling decision making process. Therefore, the traditional methods should be enhanced or replaced for modeling and describing human decision making.

Recently, quantum mechanics inspired explanation of “rational violation” is proposed and tested [11–13]. It showed that quantum explanation is able to explain and illustrate two previously mentioned violations successfully. Essentially, noncommutative property and superposition principle of quantum mechanics inspired probability theory are natural tools to explain the violations. Furthermore, the approach is also capable to produce “human-like” decision during simulation [11–14]. “Human-like” in this paper refers to that the intelligent agent is able to perform decisions similar to human in same scenario. On the other hand, previous researches did not model complex environment and decision spaces which are practical to implement on intelligent agent.

In this paper, a generalized quantum-inspired decision making model (QDM) is proposed. QDM helps to extend previous research findings and model more complicated decision space. Four experiments are concluded and verified QDM where the experiment results agree with empirical almost perfectly. The cognitive biases in decision making process are resolved in experiments. QDM is expected to help researches to model real life decision making process and improve the performance of current intelligent agent for generating “human-like” decision.

This paper is based on three hypotheses. First, because QDM is capable to explain violations of “rational decisions” of human behavior, authors believe that QDM could result “human-like” decisions. Second, all decisions in a scenario can be quantified. Third, some parameters are predefined because the paper is mainly discussing decision making model. The paper offers a preliminary result of QDM and its applications. The representation introduced in this paper has its own advantages and limitations. In future, more theoretical works of QDM are needed to be explored. An elegant representation of QDM is also required.

The paper is formatted as follows.  Section 2 presents the methodology and mathematical description.  Section 3 showed experiment results to verify the model and finally Section 4 concludes the paper and discusses future works.

## 2. Methodology

### 2.1. Environment Setting

In this section, the paper sets two types of environment for further discussion. In this paper, authors considered two players involved only in order to simplify the scenario and establish fundamental analysis of the topic.

#### 2.1.1. First Type Two Players Game

First Type Two Players Game (FTTP) contains two characters: Player 1 and Player 2. In this context, at least one of the players is an intelligent agent which is sufficient to provide and execute necessary functionalities and make decisions. Mathematically, let *𝒜* = {*a*_1_, *a*_2_,…, *a*_*N*_ | *N* ≥ 1} be the Player 1's decision space and *ℬ* = {*b*_1_, *b*_2_,…, *b*_*M*_ | *M* = 2^*m*^, *m* ≥ 1} be the Player 2's decision space. Elements of *𝒜* and *ℬ* can be formed with any semantic description: names, codes, and so on.

This type of game is used as main scene in the following sections to describe QDM.

#### 2.1.2. Second Type Two Players Game

Second Type Two Players Game (STTP) contains two players: Player 1 and Player 2. In this context, at least one of the players is an intelligent agent which is sufficient to provide and execute necessary functionalities and make decisions. Both players share same decision space *𝒟* = {*d*_1_, *d*_2_,…, *d*_*M*_ | *M* = 2^*m*^, *m* ≥ 1}. Similarly, the elements of *𝒟* can be any semantic description. During the game, Player 1 will act a decision from *𝒟*, and Player 2 have to select an appropriate decision from *𝒟* to respond.

#### 2.1.3. Payoff

Players will receive amount of rewards by performing any decision. A payoff matrix which assigns rewards to each decision for both players is defined. The payoff matrix according to two players is necessary to be produced before the game started in both FTTP and STTP. The received payoff of a player is determined by utility function or utility vector. The elements of payoff matrix are not necessarily real numbers. However, payoff has to be real number due to the limitation of Hamiltonian operator (explained in Section 2.4).

### 2.2. Two-Stage Quantum Decision Model

*Two-Stage Quantum Decision Model* assumes that Player 1 makes a decision *a*_*i*_ then Player 2 has to react an appropriate decision *b*_*j*_.

Let Ψ be the state vector and defined as Ψ = (*ψ*_1_, *ψ*_2_,…, *ψ*_*i*_,…, *ψ*_*N*_)^*T*^ where *ψ*_*i*_ = (*ψ*_*i*,1_, *ψ*_*i*,2_,…,*ψ*_*i*,*j*_,…, *ψ*_*i*,*M*_)^*T*^, *i* ∈ [1, *N*], and *j* ∈ [1, *M*] and *ψ*_*i*,*j*_ means the state of performing decision *b*_*j*_ given *a*_*i*_. Furthermore, the state vector satisfies ∑_*i*∈[1,*N*],*j*∈[1,*M*]_ | *ψ*_*i*,*j*_|^2^ = 1, and |*ψ*_*i*,*j*_|^2^ is the probability of state *ψ*_*i*,*j*_ according to quantum mechanics. A noted fact is that the order of elements does matter in this paper.

Before the game starts, set the initial state Ψ_0_ as

(1) $$\begin{array}{c} \Psi_{0}=\frac{1}{\sqrt{N\times M}}\left(\begin{array}{c} 1\\ 1\\ \vdots \\ 1\\ 1 \end{array}\right), \end{array}$$

where there are *N* × *M* elements in the state vector.

#### 2.2.1. Stage One

Assume Player 1 makes decision *a*_*i*_ and Player 2 recognized it successfully; the state vector is transformed to state Ψ_1_ that rules out other probabilities and only retains *ψ*_*i*_, and the description of Ψ_*i*_ is defined as follows:

(2) $$\begin{array}{c} \Psi_{1}=\frac{1}{\sqrt{M}}\left(\begin{array}{c} 0\\ 0\\ \vdots \\ \psi_{i}\\ \vdots \\ 0 \end{array}\right), \end{array}$$

where *ψ*_*i*_ = (1_1_, 1_2_,…, 1_*M*_)^*T*^.

#### 2.2.2. Stage Two

According to time-dependent Schrödinger equation, the time evolution is determined by (3), where *ι* is imaginary unit, defined as $\iota =\sqrt{-1}$:

(3) $$\begin{array}{c} \iota \frac{d\Psi }{dt}=H\cdot \Psi . \end{array}$$

The solution to (3) is

(4) $$\begin{array}{c} \Psi_{2}\left(t\right)=e^{-\iota \cdot t\cdot H}\cdot \Psi_{1}, \end{array}$$

where *t* ≥ 0 and Hamiltonian operator *H* is determined by the sum of two matrices: *H*_*A*_, *H*_*B*_ ∈ ℝ^(*M*×*N*)×(*M*×*N*)^:

(5) $$\begin{array}{c} H=H_{A}+H_{B}. \end{array}$$

The detailed description of Hamiltonian operator can be found in Section 2.4.

State Ψ_1_ is transformed to Ψ_2_ by employing (4) with given time *t*. Note that (4) is not a conventional representation to time-dependent Schrödinger equation. In this paper, the Plank constant *ℏ* is omitted. And authors assume that Ψ_1_ and Ψ_0_ are associated with *t* = 0.

### 2.3. One-Stage Quantum Decision Model

The previous section presented the decision making strategy based on Player 1's decision. In this section, a* one-stage quantum decision model* is described. The model in the section does not require Player 1's decision as reference and makes decision directly.

This approach will produce fuzzier result of decision-making certainly; however, it is extremely important when Player 2 is not able to collect enough evidence to perform two-stage QDM.

The concept of constructing* one-stage QDM* is same as Section 2.2.2. Equation (4) is modified to

(6) $$\begin{array}{c} \Psi_{2}\left(t\right)=e^{-\iota \cdot t\cdot H}\psi_{0}. \end{array}$$

Equation (6) indicates that the state Ψ_0_ is directly transformed to Ψ_2_ without knowing Ψ_1_ and it seems unreasonable. In fact, (6) can be viewed as the summarization of all possible Ψ_1_ due to superposition principle. The interpretation is showed as follows:

(7) Ψ2(t)=e−ι·t·H·1N(∑i∈[1,N]Ψ1i)=1N∑i∈[1,N]e−ι·t·HΨ1i,

where Ψ_1_*i*__ represents that Player 1 makes decision *a*_*i*_.

### 2.4. Hamiltonian Operator

According to quantum mechanics, Hamiltonian operator in matrix form is required to be a Hermitian matrix at least for ensuring that *e* ^−*ι*·*t*·*H*^ is a unitary operator. And due to the property of Hermitian matrix, payoff has to be real number. Hamiltonian is used to rotate the state vector to the desire basis. A suggested solution of *H*_*A*_ and *H*_*B*_ is presented.

#### 2.4.1. *H*_*A*_

*H*_*A*_ is a diagonal matrix where *H*_*A*_ = (*H*_*A*,*a*_1__, *H*_*A*,*a*_2__,…, *H*_*A*,*a*_*N*__) rotates the state to the desired decision according to Player 1's decision, and *H*_*A*,*a*_*i*__ ∈ ℝ^*M*×*M*^ is defined as a edited Hadamard transform:

(8) $$\begin{array}{c} H_{m}=\left(\begin{array}{cc} H_{m-1} & H_{m-1}\\ H_{m-1} & -H_{m-1} \end{array}\right),\;\;H_{1}=\left(\begin{array}{cc} 1 & 1\\ 1 & -1 \end{array}\right), \end{array}$$

where *m* is same as the *m* defined in Section 2.1.

An adjust matrix *U* ∈ ℝ^*M*×*M*^ is defined as

(9) $$\begin{array}{c} U=\left(\begin{array}{cccc} u_{i} & 1 & \cdots & 1\\ 1 & u_{i} & \cdots & 1\\ \vdots & \vdots & \ddots & \vdots \\ 1 & 1 & \cdots & u_{i} \end{array}\right), \end{array}$$

where *u*_*i*_ is the received payoff of Player 2 given *a*_*i*_ according to utility function or utility vector of Player 2.

Therefore, *H*_*A*,*a*_*i*__ is defined as

(10) $$\begin{array}{c} H_{A,a_{i}}=\frac{1}{\sqrt{u_{i}^{2}+2^{m}-1}}U\circ H_{m}, \end{array}$$

where $1/\sqrt{u_{i}^{2}+2^{m}-1}$ is a scalar and “∘” represents entrywise product.

#### 2.4.2. *H*_*B*_

*H*_*B*_ exists in STTP only. In FTTP, *H*_*B*_ is set as null matrix. Cognitive Science findings suggest that if Player 1 chooses an action, Player 1 would tend to think that Player 2 will choose the same decision; *H*_*B*_ ∈ ℝ^*M*^2^×*M*^2^^ is constructed in a special way. Let *H*_*B*_′ = {*H*_1_′, *H*_2_′,…, *H*_*M*_′ | *H*_*i*_′ ∈ ℝ^*M*×*M*^}.

For each *H*_*i*_′, it follows certain rules.

- *H*_*i*_′ is a symmetric matrix.
- The *i*th row and *i*th column of *H*_*i*_′ must be full of 1 s.
- Other than the *i*th row, each row contains *M*/2 positive 1 s and *M*/2 negative 1 s.

Let *H*_*B*_0__ ∈ ℝ^*M*^2^×*M*^2^^ = 0 be the initial matrix. 0 represents null matrix. Let the index of *H*_*k*_′ be (*i*_1_, *j*_1_) and the index of *H*_*B*_0__ be (*i*_2_, *j*_2_). Replace 0 in *H*_*B*_0__ as the corresponding element in *H*_*k*_′ using the following relationship:

(11) i2=(i1−1)×M+k,j2=(j1−1)×M+k.

Employing (11) to every *k* ∈ [1, *M*]. Normalizing the final product from above,

(12) $$\begin{array}{c} H_{B}=\frac{-\gamma }{\sqrt{M}}H_{B_{0}}, \end{array}$$

where $-\gamma /\sqrt{M}$ is a scalar and *γ* is a constant.

### 2.5. Payoff Matrix and Utility Function

As the paper discussed previously, payoff matrix and corresponding utility function/vector are necessary to be produced and affect the result fundamentally. Some suggestions of settings are presented in this section.

The concepts of payoff matrix and utility function/vector are borrowed from Game Theory, which are useful to represent decision space in two dimensions. Payoff matrix, for certain purposes, can be abstracted and estimated from environment. Utility function is used to calculate the expected payoff of a player. There are many ways to perform this function in Game Theory and reinforcement learning. Utility function/vector may be learned during training process. A reliable utility function/vector would increase the robustness of QDM.

Usually, payoff matrix is easy to define or estimate. On the other hand, although utility function has well definition in Game Theory, the actual received payoff is different from mathematical formalization. For example, a famous hypothesis in Game Theory is that every participant in the game is “evil.” Altruism, an important factor of humanity, on the contrary, is rarely mentioned. Involving “altruistic” factor to adjust utility function may help model produce more “human-like” decision.

## 3. Experiment Results

### 3.1. Prisoner's Dilemma

Prisoner's Dilemma is a canonical Game Theory problem which has been used in discussing and analyzing human behavior and decision making. The payoff matrix is described in Table 1. The Nash Equilibrium suggests that both parties have to defect in standard Game Theory. However, empirical studies argue differently.

Table 2 presented several well-known empirical studies on Prisoner's Dilemma. By employing proposed model, experiment result showed that quantum-inspired decision making model matches Prisoner's Dilemma almost perfectly.

The experiment is set as follows.

(i) The state vector Ψ = (*ψ*_1,1_, *ψ*_1,2_, *ψ*_2,1_, *ψ*_2,2_)^*T*^, where 1 represents “defect” and 2 represents “cooperate.”
(ii) Initial state Ψ_0_ = 1/2(1,1, 1,1)^*T*^.
(iii) If Player 1 chooses “defect,” the state vector changes to $\Psi_{1}=1/\sqrt{2}{\left(1,1,0,0\right)}^{T}$; if Player 1 chooses “cooperate,” the state vector changes to $\Psi_{1}=1/\sqrt{2}{\left(0,0,1,1\right)}^{T}$.
(iv) Rotation matrix *H* is equal to
  (13) H=11+u2(u1001−u0000u1001−u) +−γ2(10100−10110−100101).
(v) Set *u* = 0.51 and *γ* = 2.09 with *t* = *π*/2 [11].

By performing the settings above, the experiment concludes the following result.

- By known Player 1 choosing “defect,” the probability vector is (0.4505,0.1556,0.3552,0.3887) for the (*p*_1,1_, *p*_1,2_, *p*_2,1_, *p*_2,2_) where *p*_*i*,*j*_ indicates the probability of choosing *j* according to *i*.
- By known Player 1 choosing “cooperate,” the probability vector is (0.0904,0.3034,0.5583,0.0478) for the (*p*_1,1_, *p*_1,2_, *p*_2,1_, *p*_2,2_).
- By unknown Player 1's decision, the probability vector is (0.5653,0.4347) for the (*p*_1_, *p*_2_).

Therefore, the probability of Player 2's decision, in this case, “defect,” is (0.8052,0.6483,0.5653) for the (known “defect”, known “cooperate”, unknown). The average result of empirical studies is (0.84,0.66,0.55). The model produced the similar result to average result of empirical studies in Table 2.

### 3.2. Splitting Money Game

Splitting Money Game is also a frequently used example in Game Theory. The game is described as follows. You and your friend are splitting 7 dollars. Your friend makes an offer to you from 0 dollar to 7 dollars, such as 3 dollars or 5 dollars. If you accept the offer, then you will receive such dollars, and your friend will take the rest. However, if you reject the offer, you and your friend both will receive nothing, and the money will be donated. The payoff matrix is showed in Table 3.

An online anonymous survey of this game has been conducted and received 302 respondents. The result is showed in Table 4.

The experiment is set as follows.

(i) The state vector Ψ = (*ψ*_1,1_, *ψ*_1,2_, *ψ*_2,1_,…, *ψ*_8,2_)^*T*^, where in *ψ*_*i*,*j*_, *i* represents offer (*i* − 1)$, and *j* represents “accept”(1) or “reject”(2).
(ii) Initial state Ψ_0_ = 1/4(1,1,…, 1)^*T*^.
(iii) Stage vector for offer *i* is $\Psi_{1}=1/\sqrt{2}{\left(0,0,\ldots ,1,1,\ldots ,0\right)}^{T}$.
(iv) Rotation matrix *H* is diagonal matrix (*H*_*A*,1_,…, *H*_*A*,8_):
  (14) $$\begin{array}{c} H_{A,i}=\frac{1}{\sqrt{1+u_{i}^{2}}}\left(\begin{array}{cc} u_{i} & 1\\ 1 & -u_{i} \end{array}\right). \end{array}$$
(v) Set utility vector as **u** = (−0.41, −0.39, −0.32,0.19,0.38,0.11,0.05,0.04) where the elements of **u** are corresponding to *u*_*i*_ in order and *t* = *π*/2.

By performing the setting above, the experiment concludes the following results.

- For known different offers, experiment produces a probability vector for choosing “accept” by Player 2: (0.1490,0.1615,0.2097,0.6834,0.8321,0.6087,0.5499,0.5399).
- For known different offers, experiment produces a probability vector for choosing “reject” by Player 2: (0.8510,0.8385,0.7903,0.3166,0.1679,0.3913,0.4501,0.4601).
- For unknown offer, the probability vector is (0.4668,0.5332) for choosing “accept” and “reject,” respectively.

### 3.3. The Price Is Right?

The Price is Right is a game where participants need to choose the same price as opponent's choice in order to win. The description of the game is given as follows. Las Vegas proposed a new game. The dealer will give you four cards, and each card has a price on it; for example, card 1 is 1000$, card 2 is 2000$, and so on. Before the game starts, dealer would write down a price from one of the cards secretly and then save it in an envelope; witness would make sure nobody can touch the envelope during the game. Now the game started; you need to choose one of the cards. After you made your choice, witness will open the envelope and dealer will judge the result according to the following rules.

1. If the price of the card you chose is same as the price which is written, you win the such amount of money. For example, you choose a card with 1000$, and dealer also wrote 1000$. You will win 1000$.
2. If the price of the card you chose is different from the price that is written, you will lose. And you will be judged as loser, and you need to pay half of the difference. For example, you choose the card with 1000$, but the dealer wrote 4000$ instead, then you need to pay (4000 − 1000)/2 = 1500$ to the dealer.

The payoff matrix of The Price is Right is presented in Table 5. An online anonymous survey of this game has been conducted and received 72 respondents. The result is showed in Table 6.

The experiment is set as follows.

(i) The state vector Ψ = (*ψ*_1,1_, *ψ*_1,2_, *ψ*_1,3_, *ψ*_1,4_,…, *ψ*_4,4_)^*T*^, where in *ψ*_*i*,*j*_.
(ii) Initial state Ψ_0_ = 1/4(1,1,…, 1)^*T*^.
(iii) First stage vector for different *i* is Ψ_1_ = 1/2(0,0,…, 1,1, 1,1 …, 0).
(iv) Rotation matrix *H* is sum of diagonal matrix *H*_*A*_ = (*H*_*A*,1_,…, *H*_*A*,4_) and *H*_*B*_.
  (a) For *H*_*A*_,
    (15) $$\begin{array}{c} H_{A,i}=\frac{1}{\sqrt{1+u_{i}^{2}}}\left(\begin{array}{cc} u_{i} & 1\\ 1 & -u_{i} \end{array}\right). \end{array}$$
  (b) For *H*_*B*_,
    (16) H1′(111111−1−11−11−11−1−11),H2′(−111−1111111−1−1−11−11),H3′(1−11−1−111−11111−1−111),H4′(1−1−11−11−11−1−1111111).
(v) Set utility vector as **u** = (0.25, −2.225, −7,5) where the elements of **u** are corresponding to *u*_*i*_ in order *γ* = 2.09 and *t* = *π*/2.

By performing the setting above, the experiment concludes the following results.

- For choosing 1000$ : 0.1512.
- For choosing 2000$ : 0.1664.
- For choosing 3000$ : 0.4572.
- For choosing 4000$ : 0.2252.

### 3.4. A Sheriff's Dilemma

A Sheriff's Dilemma is a classic Bayesian Game in Game Theory. A Bayesian Game introduces multiple payoff matrices with corresponding probability to describe the scenario. The description of the game is presented as follows. You, the sheriff, are facing a suspect. The suspect has a gun. You are pointing at each other, and now, you need to make the decision whether you are going to shoot him (assume there is no way to talk). The suspect has a possibility to be the criminal, but also can be innocent. Here, let us say it is half and half, which means that you cannot really tell whether the suspect is a criminal or innocent. The criminal would rather shoot even if the sheriff does not, as the criminal would be caught if he does not shoot. The innocent suspect would rather not shoot even if the sheriff shoots. The payoff matrix is presented in Table 7.

An online anonymous survey of this game has been conducted and received 89 respondents. The result is showed in Table 8.

The experiment is set as follows.

(i) The state vector Ψ = (*ψ*_1,1_, *ψ*_1,2_, *ψ*_2,1_,…, *ψ*_4,2_)^*T*^, where in *ψ*_*i*,*j*_.
  (a) When *i* = 1,2, 1 represents “shoot” and 2 represents “not shoot” when suspect is innocent.
  (b) When *i* = 3,4, 3 represents “shoot” and 4 represents “not shoot” when suspect is a criminal.
  (c) When *j* = 1,2, 1 represents “shoot” and 2 represents “not shoot” for sheriff.
(ii) Initial state $\Psi_{0}=1/2\sqrt{2}{\left(1,1,\ldots ,1\right)}^{T}$.
(iii) First stage vector for different *i* is $\Psi_{1}=1/\sqrt{2}(0,0,\ldots ,1,1,\ldots ,0)$.
(iv) Rotation matrix *H* is a diagonal matrix (*H*_*A*,1_,…, *H*_*A*,4_):
  (17) $$\begin{array}{c} H_{A,i}=\frac{1}{\sqrt{1+u_{i}^{2}}}\left(\begin{array}{cc} u_{i} & 1\\ 1 & -u_{i} \end{array}\right). \end{array}$$
(v) Set utility vector as **u** = (0.3, −0.29,0.7, −0.15) where the elements of **u** are corresponding to *u*_*i*_ in order and *t* = *π*/2.

By performing the setting above, the experiment concludes the following results.

- For known suspect shoot, experiment produces a probability for choosing shoot by sheriff which is 0.8752.
- For known suspect not shoot, experiment produces a probability vector for choosing shoot by sheriff which is 0.2929.
- For unknown suspect shoot/not shoot, experiment produces a probability vector for choosing shoot by sheriff which is 0.5827.

## 4. Conclusions and Future Works

This paper introduced a generalized quantum-inspired decision making model for intelligent agent. And the proposed model is verified by four experiments successfully. The model is aiming to provide a tool for intelligent agent to perform “human-like” decision instead of “machine-like” decision. Even though this paper limits the setting between two players, two-dimensional decision spaces are in fact the foundation of multiagents environment. Furthermore, the presented model is able to model much more complex and larger decision space than previous researches.

Some future works are considered. The first problem is that the number of decisions does not always follow 2^*m*^, and how to disable one or more necessary dimensions is fairly important to study. Second, since the payoff matrix and utility function affect the results fundamentally, the study of both may improve the performance of the model. Third, more social studies and empirical results on human decision making are needed; they are used for adjusting and improving the model. Fourth, the performance of the model in multiagents environment is worthy of being studied.

## Figures and Tables

**Table 1:** Payoff matrix of Prisoner's Dilemma.

	Your defect	Your cooperate
Other defects	Other: 10, You: 10	Other: 25, You: 5
Other cooperate	Other: 5, You: 25	Other: 20, You: 20

**Table 2:** Empirical studies and experiment results using QDM on Prisoner's Dilemma (the probability indicates that Player 2 chooses “defect” by known “defect,” “cooperate,” or “unknown”).

	Known defect	Known cooperate	Unknown
Shafir and Tversky [3]	97%	84%	63%
Li and Taplin [4]	83%	66%	60%
Croson [15]	67%	32%	30%
Buesmeyer et al. [16]	91%	84%	66%
Average of above	**84% **	**66% **	**55% **
QDM	81%	65%	57%

**Table 3:** Payoff matrix of Splitting Money Game.

	Offer							
	0$	1$	2$	3$	4$	5$	6$	7$
Accept	You: 0$	You: 1$	You: 2$	You: 3$	You: 4$	You: 5$	You: 6$	You: 7$
	Other: 7$	Other: 6$	Other: 5$	Other: 4$	Other: 3$	Other: 2$	Other: 1$	Other: 0$

Reject	You: 0$	You: 0$	You: 0$	You: 0$	You: 0$	You: 0$	You: 0$	You: 0$
	Other: 0$	Other: 0$	Other: 0$	Other: 0$	Other: 0$	Other: 0$	Other: 0$	Other: 0$

**Table 4:** The Game Theory prediction, survey result, and experiment results using QDM on Splitting Money Game.

	Offer								
	0$	1$	2$	3$	4$	5$	6$	7$	Unknown
Game Theory accept	100%	100%	100%	100%	100%	100%	100%	100%	100%
Game Theory reject	0%	0%	0%	0%	0%	0%	0%	0%	0%
Survey accept	15.19%	16.19%	21.28%	68.42%	82.58%	61.07%	54.96%	54.42%	46.90%
Survey reject	84.81%	83.81%	78.72%	31.58%	17.42%	38.93%	45.04%	45.58%	53.10%
QDM accept	14.90%	16.15%	20.97%	68.34%	83.21%	60.87%	54.99%	53.99%	46.68%
QDM reject	85.10%	83.85%	79.03%	31.66%	16.79%	39.13%	45.01%	46.01%	53.32%

**Table 5:** Payoff matrix of The Price is Right.

Offer	1000$	2000$	3000$	4000$
1000$	You: 1000$	You: −500$	You: −1000$	You: −1500$
	Dealer: −1000$	Dealer: 500$	Dealer: 1000$	Dealer: 1500$

2000$	You: −500$	You: 2000$	You: −500$	You: −1000$
	Dealer: 500$	Dealer: −2000$	Dealer: 500$	Dealer: 1000$

3000$	You: −1000$	You: −500$	You: 3000$	You: −500$
	Dealer: 1000$	Dealer: 500$	Dealer: −3000$	Dealer: 500$

4000$	You: −1500$	You: −1000$	You: −500$	You: 4000$
	Dealer: 1500$	Dealer: 1000$	Dealer: 500$	Dealer: −4000$

**Table 6:** The Game Theory prediction, survey result, and experiment results using QDM on The Price is Right.

	Offer			
	1000$	2000$	3000$	4000$
Survey's choice	13.89%	20.83%	44.44%	20.83%
QDM choice	15.12%	16.64%	45.72%	22.52%

**Table 7:** Payoff matrix of A Sheriff's Dilemma.

If the suspect is innocent			If the suspect is a criminal		
	Shoot	Not Shoot		Shoot	Not Shoot
Shoot	You: −3	You: −1	Shoot	You: 0	You: 2
	Suspect: −1	Suspect: −2		Suspect: 0	Suspect: −2

Not Shoot	You: −2	You: 0	Not Shoot	You: −2	You: −1
	Suspect: −1	Suspect: 0		Suspect: −1	Suspect: −1

*The row is sheriff's decisions; column is suspect's decisions.

**Table 8:** The Game Theory prediction, survey result, and experiment results using QDM on A Sheriff's Dilemma.

	Known suspect shoot	Known suspect not shoot	Unknown suspect shoot/not shoot
Survey shoot	88.76%	26.97%	61.80%
Survey not shoot	11.24%	73.03%	38.20%
QDM shoot	87.52%	29.29%	58.27%
QDM not shoot	12.48%	70.71%	41.73%
